# Association between healthy lifestyle score and breast cancer

**DOI:** 10.1186/s12937-020-0520-9

**Published:** 2020-01-14

**Authors:** Batoul Ghosn, Sanaz Benisi-Kohansal, Soraiya Ebrahimpour-Koujan, Leila Azadbakht, Ahmad Esmaillzadeh

**Affiliations:** 1 Department of Community Nutrition, School of Nutritional Sciences and Dietetics, Tehran University of Medical Sciences, P.O. Box 14155-6117, Tehran, Iran; 2 Obesity and Eating Habits Research Center, Endocrinology and Metabolism Molecular- Cellular Sciences Institute, Tehran University of Medical Sciences, Tehran, Iran; 3 Department of Community Nutrition, School of Nutrition and Food Science, Isfahan University of Medical Sciences, Isfahan, Iran

**Keywords:** Diet, Physical activity, Smoking, Lifestyle, Breast cancer

## Abstract

**Background:**

Majority of earlier studies have assessed the association between individual lifestyle factors and the risk of breast cancer (BC); however, limited information is available linking the whole lifestyle factors to BC. We aimed to examine the association between combined lifestyle score (diet, physical activity (PA) and smoking) and risk of BC in Iranian population.

**Methods:**

This population-based case-control study included 350 newly diagnosed cases of BC and 700 healthy controls randomly selected from adult women. Dietary intakes, PA and smoking status of study participants were examined using validated questionnaires. The lifestyle risk factors examined in this study included cigarette smoking, physical inactivity, and Healthy Eating Index-2010 (HEI-2010). The lifestyle score ranged from zero (non-healthy) to 3 (most healthy) points. Logistic regression models were fitted to investigate the association between combined lifestyle scores and odds of BC.

**Results:**

Mean age and body mass index (BMI) of study participants were 62.4 years and 24.3 kg/m^2^, respectively. In the whole study population, individuals with the highest healthy lifestyle score (HLS) were 0.38 times less likely to have BC than those with the lowest score (OR: 0.62; 95% CI: 0.40, 0.93, *P*_*trend*_ = 0.01). The analysis by menopausal status showed that postmenopausal women with the highest HLS had 44% lower odds of BC compared with those with the lowest score (OR: 0.56; 95% CI: 0.36, 0.88, *P*_*trend*_ = 0.004). Such association was not seen in premenopausal women. After analyzing each component of HLS, we found that individuals with the highest HEI score were 46% less likely to have BC than those with the lowest score (OR: 0.54; 95% CI: 0.35, 0.82, *P*_*trend*_ <  0.001). No other significant associations were found between PA and smoking and risk of BC.

**Conclusions:**

Significant inverse associations were found between HLS and HEI with BC especially among postmenopausal women. Prospective studies are required to confirm these findings.

## Introduction

Breast cancer (BC) is highly prevalent in the world, particularly in developing countries. Incidence rates differ widely throughout the world, from 27 per 100,000 in Middle Africa and Eastern Asia to 92 per 100,000 in Northern America [1]. In Iran, BC is the fifth most common causes of death related to cancer comprising 24.4% of all cancers with age standardized rate (ASR) of 23.1 per 100,000 [2]. The national database revealed a progressive increasing trend in the cumulative probability of BC incidence for individuals aged 15–79 years in Iran in the last 30 years [3].

Several modifiable risk factors including overweight or obesity [4], alcohol use [5], physical inactivity [6] and prolonged steroid hormones exposure [7] have been linked to BC. Non-modifiable risk factors include increasing age [8], positive family history of BC [9] and reproductive factors [8]. There is currently limited evidence that diet is related to BC. In a review on prospective epidemiologic studies, Michels et al. failed to find any significant association between diet and BC [10]. However, the American Cancer Society recommendations regarding healthy lifestyle include eating a healthy diet, getting at least 150 min of moderate intensity exercise each week, avoiding smoking and keeping low stress levels [11]. Thus, to reduce the risk, it is important to determine the degree to which women are adhering to these recommendations.

There are few studies published on the link between healthy lifestyle index and BC. Arthur et al. found a 30% higher risk of BC among those in the lowest category (0–10 points) of the healthy lifestyle score (HLS) compared to those in the highest category (≥15 points) [12]. Similarly, Sanchez-Zamorano et al. concluded that healthy lifestyle was associated with a decreased risk of having BC [13]. However, some other studies failed to find any association [14]. It must be kept in mind that these studies were conducted in western countries and to our knowledge, no study was done in eastern countries, specifically Iran, investigating the healthy lifestyle factors in relation to risk of BC. Given the limitations in the construction of HLS, inconsistent findings and small number of cases in previous studies, generalization of their findings might be limited.

We examined the contribution of whole lifestyle to the risk of BC because of these important factors: (1) the different pattern of diet and physical activity (PA) in developing countries compared with those in western nations, (2) BC being the most prevalent cancer among Iranian women. Therefore, this study was done to assess the association between healthy lifestyle factors in relation to the risk of BC among Iranian adult women.

## Methods

### Research design and methods

This project is a population-based case-control study on women aged above 30 years. Patients with BC were recruited if disease status was diagnosed during the maximum of last six-month by physical examination and mammography findings. Patients were recruited from those referred to hospitals or private clinics who were undergoing surgical resection of BC, chemotherapy or radiotherapy, or all of them. BC was defined as primary incident breast tumor with invasive behavior which its histology was available from medical registered history. We did not include patients with a history of any type of neoplastic lesion or cysts (exception of current BC) as well as those with a history of any hormone replacement therapy. As hormone use is not part of lifestyle, we did not consider it in our lifestyle score construction. In addition, those who were on a special diet were not included in this study. Age-matched controls were selected from healthy women, who have no relationship with BC patients or had no family history of BC. In addition to age, we did our best to match controls in terms of socioeconomic status (SES) with the cases. Controls meeting our inclusion criteria (female, Iranian nationality, no history of any malignancy, cysts and medical disorder, having no special diet or hormone replacement therapy) were selected. Controls were randomly selected from apparently healthy women by multistage cluster random sampling method. Individuals who were not relatives of patients with BC that attended primary health care centers for their annual personal checkup or attended to receive required information about their children (i.e. growth monitoring, vaccination, …) were selected. From several healthcare centers in Isfahan, we randomly chose two centers. First, considering the population under coverage, and then based on attendance of women to these centers, required sample were recruited. Eligible subjects culminated to 350 cases and 700 controls. Written informed consents were obtained from all subjects.

### Assessment of diet

Dietary data was collected using a 106-item Willett-format semi-quantitative dish-based food frequency questionnaire (FFQ) which was designed and validated specifically for Iranian adults. We computed daily intakes of all food items and then converted them to grams per day using household measures [15]. The validity of the questionnaire as well as the detailed information about design and foods included has been reported previously [16]. Briefly, it is based on frequency of consumption of food along the last year in addition to common portion sizes in Iran. The daily value for each item was calculated according to food composition, average of reported frequency, and specified portion size. As for nutrient intakes, it was calculated by adding the nutrient contents of all food and dishes. The nutrient intake for each participant was obtained by Nutritionist IV software, which was modified for Iranian foods. In general, FFQ provides valid and reliable measures of the average intake of foods [17], food groups [17], and nutrients [18] over the long-term.

### Assessment of physical activity

According to an interview-based International Physical activity questionnaire, data on PA was obtained by using participants oral responses, and is expressed as metabolic equivalent h/wk. (MET h/wk) [19]. The questionnaire included queries on five activity domains: job-related PA; transportation PA; activities for housework, and house maintenance; recreation, sports, and leisure-time PA; and time spent sitting. We asked participants to consider all the vigorous and moderate activities as well as the time spent during the last seven days.

### Assessment of smoking

Smoking was examined using a pretested self-administered questionnaire. The question to the participants was “Are you a smoker or not a smoker or an ex-smoker?”. Since the prevalence of smoking in our study population was low, we assumed participants as “smokers” or “nonsmokers”. In the current study, participants who have reported smoking were defined as smokers, and those who reported as nonsmokers or ex-smokers were considered as “nonsmokers”.

### Assessment of healthy lifestyle score

**Fig. 1:**
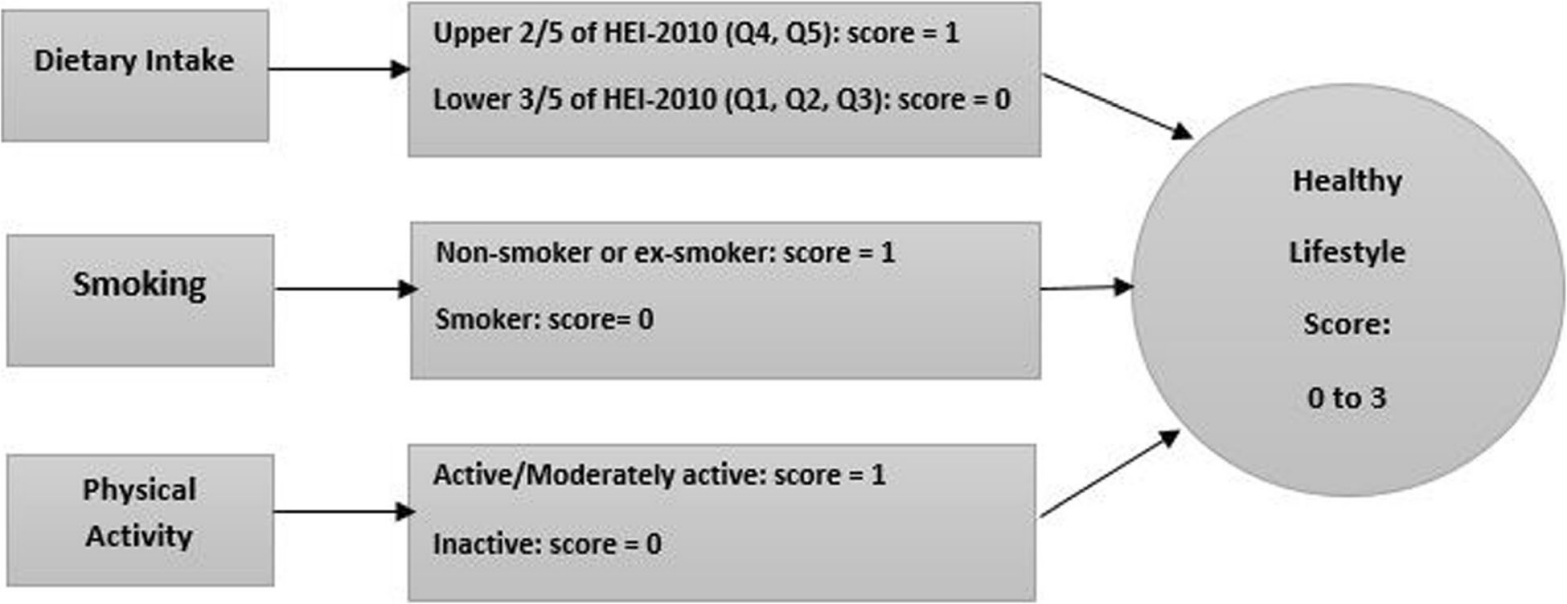
Healthy lifestyle score construction

To construct a healthy lifestyle score, we used data from dietary intakes, PA and smoking status**.** For the assessment of a healthy dietary intake, we used the previously designed HEI-2010 [20]. The index is constructed of 12 components (total and whole fruits, total vegetables, greens and beans, whole grains, dairy, total protein foods, seafood and plant proteins, fatty acids, refined grains, sodium, and empty calories). Alcohol consumption was not involved in our study due to the absence of information in the original dataset. In the construction of index, first we calculated the energy adjusted intakes of the HEI-2010 components by the residual method [20]. Second, based on the deciles categories of energy adjusted intakes of these components, classification of participants was performed. The usage of decile categories of components instead of quantitative classifications was considered since scoring by deciles would be least disposed to misclassification. Participants in the highest deciles of fruits, vegetables, whole grains, nuts and legumes, long chain omega-3 fats and polyunsaturated fatty acids were given the score of 10, whereas those in the lowest deciles of these items were given the score of 1. Participants in the other deciles of these components were given the corresponding scores. Concerning sugar sweetened drinks and fruit juice, red and processed meat, trans fat, sodium intake, added sugars and saturated fatty acids, the lowest deciles were given a score of 10, whereas the highest deciles were given the score of 1. Individuals in deciles 9, 8, 7, 6, 5, 4, 3 and 2 of these components were given the scores of 2, 3, 4, 5, 6, 7, 8 and 9, respectively. Then to calculate the HEI-2010, we summed up the scores for the individual items, resulting in a minimum score of 10 and a maximum score of 100. Individuals who were in the upper two fifths of HEI-2010 were considered to have a healthy diet. As for PA, low risk groups were defined as participants with active and moderately active lifestyle. Regarding cigarette smoking, low risk groups were defined as ex-smokers and individuals who never smoked. As for the HLS development, participants were scored on three modifiable lifestyle factors as unhealthy (zero points) or healthy (one point). Participants obtained one point for each respective lifestyle factor: nonsmoking, adherence to healthy dietary intake, or regular PA. A combined score (zero to three points) was obtained by summing up the scores of the three factors (Fig. 1).

### Assessment of other variables

To measure SES of participants, the socio-economic questionnaire constituting of categorized items on three groups of low, middle and high SES was completed. Also, we evaluated the anthropometric indices of subjects. Measurement of weight was done by digital scales while subjects were minimally clothed and not wearing shoes. Weight was recorded to the nearest 100 g. Height was assessed by using a tape measure while the subjects are not wearing shoes, standing, and shoulders in normal position. By the help of a self-reported questionnaire, we obtained the data on weight (in kilograms) and height (in centimeters). Body mass index (BMI) was calculated by dividing the weight in kilograms by square of height in meters. Participants were classified into two groups based on their BMI: normal weight (18.5–24.9 kg/m^2^), overweight or obese (> 25.0 kg/m^2^). Waist circumference was taken at the narrowest level, and hip circumference was measured at the maximum level over light clothing by the aid of an unstretched tape measure without any pressure to body surface. Measurements were calculated to the nearest 0.1 cm.

### Statistical analysis

Before starting the analysis, we found that number of individuals in the lowest category of HLS (i.e. HLS = 0) was very low (*n* = 22). To avoid having wide confidence intervals (CIs) in this category, we decided to merge the lowest two categories of HLS (0 and 1) and consider them as one category. General characteristics and dietary intakes of cases and controls were assessed using independent samples Student’s t test for continuous variables and chi-square for categorical variables. General characteristics and dietary intakes of study participants across categories of the HLS were examined using one-way analysis of variance (ANOVA) for continuous variables and chi-square for categorical variables. The association of HLS with BC was assessed by using conditional logistic regression in different models. Age (continuous), residence (urban/rural), marital status (non/married/not married), SES (poor/middle/high class), education (educated/not educated), family history of BC (yes/no), menopausal status (yes/no), breast feeding (yes/no), history of disease (yes/no) and supplement use (yes/no) were adjusted in the first model. BMI was additionally adjusted in the second model. To determine the association of individual components of HLS with BC risk, we constructed crude and multivariable-adjusted models controlling for above mentioned covariates, as well as other components of HLS for each component. In addition to the whole study population, the analyses were also stratified by menopausal status. In these analyses, all above-mentioned covariates were considered except for menopausal status. All confounders were chosen based on previous publications. The statistical analyses were carried out by using IBM SPSS statistics 25. Significance level was considered at *P* <  0.05.

## Results

### General characteristics of study participants

General characteristics of study participants with and without BC are presented in Table 1. Participants with BC were more likely to be older, have a family history of BC, and menopausal than their controls. In contrary, they were less likely to be married, educated, and had a lower mean BMI than controls. The prevalence of smoking was higher among patients with BC than controls. No other significant difference was seen in terms of other general characteristics comparing individuals with and without BC.

**Table 1:** General characteristics of study participants

	Groups			Healthy Lifestyle Score			
	Cases *n* = 350	Controls *n* = 700	P^*^	1 *n* = 185	2 *n* = 455	3 *n* = 410	P^*^
Age (year)	65 ± 11	61 ± 10	< 0.001	64 ± 11	62 ± 10	62 ± 11	0.065
Residing in Urban Region	126 (36)	252 (36)	0.964	44 (24)	150 (33)	184 (45)	< 0.001
Married	262 (75)	616 (88)	< 0.001	153 (83)	382 (84)	340 (83)	0.650
Educated	59 (17)	203 (29)	< 0.001	27 (15)	100 (22)	135 (33)	< 0.001
Family History of BC	31 (9)	21 (3)	< 0.001	16 (9)	22 (5)	16 (4)	0.079
Smoker	59 (17)	91 (13)	0.055	86 (47)	63 (14)	0	< 0.001
Post-menopause	308 (88)	539 (77)	< 0.001	151 (82)	368 (81)	328 (80)	0.930
BMI (kg/m^2^)	22 ± 5	26 ± 5	< 0.001	3 ± 5	24 ± 5	25 ± 5	< 0.001
Physical Activity (MET-hr/wk)	35 ± 7	35 ± 7	0.196	30 ± 6	34 ± 7	38 ± 5	< 0.001
Breast Feeding	119 (34)	238 (34)	0.926	61 (33)	145 (32)	151 (37)	0.298
Poor Social Economic Status	115 (33)	203 (29)	0.311	79 (43)	150 (33)	90 (22)	< 0.001
History of Disease	35 (10)	63 (9)	0.407	18 (10)	50 (11)	24 (6)	0.026
Supplement Use	31 (9)	70 (10)	0.715	20 (11)	45 (10)	36 (9)	0.575

Variables: Age, BMI and PA are Means ± SD, other variable values are counts, values between () are %.

* *P* values were obtained from independent Student’s t test, one-way ANOVA or χ2 test, where appropriate

As for the distribution of participants in terms of general characteristics across categories of HLS, subjects with the highest score were more likely to be residing in urban region, educated, have a higher BMI and physically active, and less likely to have poor SES than those with the lowest score. In the highest HLS, no subjects were smokers versus 47% in the lowest. No other significant differences were found between categories of HLS in terms of other general characteristics (Table 1).

### Dietary intakes of study participants

Dietary intakes of study participants with and without BC across categories of HLS are provided in Table 2. Compared to controls, individuals with BC had higher intakes of total energy, carbohydrates, fats, saturated fats, monounsaturated fatty acids (MUFAs), trans fats, cholesterol, red and processed meat, salt, sugar sweetened beverage, and lower intakes of polyunsaturated fatty acids (PUFAs). On micronutrient level, individuals with BC had higher intakes of vitamin E, vitamin C, potassium, calcium, zinc, iron and magnesium. On HEI-2010 food groups level, individuals with BC had higher intakes of empty calories, whole fruits and dairy. In contrast, they had less intakes fruit juice, total vegetables, legumes, and lower FA ratio. A marginally significant association was found between BC and higher intakes of sea food, eggs, and vitamin B12 with no difference in vitamin B6 intake between cases and controls or copper intake.

**Table 2:** Dietary intakes of study participants

	Groups			Healthy Lifestyle Score			
	Cases (*n* = 350)	Controls (*n* = 700)		1 (n = 185)	2 (*n* = 455)	3(*n* = 410)	
	Mean ± SD	Mean ± SD	P*	Mean ± SD	Mean ± SD	Mean ± SD	P*
Total energy (kcal/d)	2495 ± 793	2178 ± 608	< 0.001	2196.9 ± 700.1	2288 ± 721	2321 ± 653	0.126
Micronutrients							
Vitamin A (IU/d)	3277 ± 2941	3093 ± 2555	0.297	2160 ± 1731	2968 ± 2534	3810 ± 3027	< 0.001
Vitamin D (IU/d)	53 ± 242	33 ± 51	0.145	21 ± 27	50 ± 216	38 ± 47	0.078
Vitamin E (IU/d)	7 ± 3	6 ± 3	0.008	6 ± 3	6 ± 3	7 ± 4	< 0.001
Vitamin C (mg/d)	69 ± 45	58 ± 38	< 0.001	49 ± 37	59 ± 38	71 ± 43	< 0.001
Vitamin B_6_ (mg/d)	2 ± 1	2 ± 0.5	0.052	1 ± 0.5	1.6 ± 0.5	1.7 ± 0.5	< 0.001
Folate (mcg/d)	601 ± 206	580 ± 189	0.103	560 ± 180	585 ± 202	602 ± 191	0.053
Vitamin B_12_ (mcg/d)	3 ± 3	2.6 ± 2	0.056	2 ± 3	3 ± 2	3 ± 2	0.001
Potassium (mg/d)	3071 ± 1128	2850 ± 818	0.001	2669 ± 882	2895 ± 990	3069 ± 876	< 0.001
Calcium (mg/d)	821 ± 331	736 ± 276	< 0.001	643 ± 276	761 ± 309	824 ± 279	< 0.001
Zinc (mg/d)	10.5 ± 3	10 ± 3	0.017	9 ± 3	10 ± 3	11 ± 3	< 0.001
Copper (mg/d)	2 ± 1	2 ± 0.5	0.086	1.6 ± 1	1.7 ± 1	1.8 ± 0.5	0.002
Selenium (mcg/d)	148 ± 52	146 ± 52	0.493	135 ± 51	145 ± 54	153 ± 51	< 0.001
Iron (mg/d)	18 ± 6	17 ± 5	0.009	16 ± 5	17 ± 6	18 ± 5	0.001
Magnesium (mg/d)	472 ± 156	442 ± 141	0.002	428 ± 145	449 ± 153	466 ± 139	0.010
Food Groups							
Carbohydrates (g/d)	340 ± 123	306 ± 97	< 0.001	318 ± 112	317 ± 113	317 ± 99	0.994
Proteins (g/d)	79 ± 30	77 ± 28	0.381	66 ± 25	77 ± 31	84 ± 26	< 0.001
Red and processed meat (g/d)	13 ± 201	10 ± 12	0.002	8 ± 10	11 ± 15	12 ± 18	0.006
White meat (g/d)	71 ± 90	74 ± 68	0.478	52 ± 59	72 ± 89	84 ± 63	< 0.001
Egg (g/d)	13 ± 19	10 ± 12	0.050	10 ± 17	10 ± 15	12 ± 14	0.097
Salt (g/d)	3.5 ± 3	2.7 ± 2	< 0.001	3.5 ± 3	3.1 ± 3	2.6 ± 2	< 0.001
Fats (g/d)	99 ± 42	78 ± 28	< 0.001	80 ± 35	85 ± 37	86 ± 32	0.109
Saturated fats (g/d)	44 ± 33	26 ± 20	< 0.001	35 ± 23	34 ± 30	28 ± 23	< 0.001
Monounsaturated fats (MUFAs) (g/d)	21 ± 9	20 ± 8	0.011	18 ± 8	20 ± 8	22 ± 8	< 0.001
Polyunsaturated fats (PUFAs) (g/d)	9 ± 4	10 ± 8	< 0.001	8 ± 5	9 ± 5	12 ± 9	< 0.001
Trans FA (g/d)	0.3 ± 0.3	0.2 ± 0.2	< 0.001	0.5 ± 0.3	0.4 ± 0.3	0.4 ± 0.3	0.004
Cholesterol (mg/d)	205 ± 134	182 ± 97	0.005	152 ± 105	184 ± 113	212 ± 107	< 0.001
Sugar sweetened beverages (g/d)	40 ± 83	22 ± 28	< 0.001	33 ± 63	28 ± 52	25 ± 52	0.265
Total fiber (g/d)	23 ± 8	22 ± 8	0.328	20 ± 7	22 ± 8	24 ± 8	< 0.001
HEI-2010 Food Groups							
Fruit Juice (g/d)	1 ± 3	2 ± 4	0.002	1 ± 1	1 ± 3	2 ± 4	< 0.001
Whole Fruit (g/d)	215 ± 195	140 ± 122	< 0.001	133 ± 148	154 ± 146	191 ± 162	< 0.001
Total Vegetables (g/d)	69 ± 67	87 ± 75	< 0.001	53 ± 43	75 ± 76	99 ± 75	< 0.001
Sea food (g/d)	12 ± 51	6 ± 12	0.066	3 ± 7	10 ± 44	8 ± 17	0.023
Total Protein (g/d)	85 ± 74	89 ± 69	0.430	69 ± 62	84 ± 77	101 ± 65	< 0.001
Legumes (g/d)	13 ± 13	15 ± 16	0.032	11 ± 9	14 ± 12	17 ± 19	< 0.001
Dairy (g/d)	257 ± 174	219 ± 143	< 0.001	177 ± 140	234 ± 166	253 ± 143	< 0.001
Whole grains (g/d)	325 ± 150	313 ± 157	0.197	311 ± 150	313 ± 157	323 ± 154	0.563
Refined grains (g/d)	115 ± 86	115 ± 74	0.887	115 ± 71	120 ± 83	109 ± 76	0.151
Sodium (mg/d)	4979 ± 1867	4795 ± 1711	0.123	4839 ± 1758	4834 ± 1753	4890 ± 1787	0.889
FA ratio	1 ± 0.4	1 ± 0.5	< 0.001	1 ± 0.4	1 ± 0.4	1 ± 1	< 0.001
Empty calories (kcal/d)	149 ± 132	83 ± 56	< 0.001	127 ± 124	106 ± 88	95 ± 84	0.001

*Obtained by ANOVA

*Ratio of poly- and monounsaturated fatty acids (PUFAs and MUFAs) to saturated fatty acids (SFAs)

Comparison of participants according to their dietary intakes across categories of HLS is shown in Table 2. On macronutrient level, individuals in the highest category of HLS had higher intake of MUFAs, PUFAs, cholesterol, total fiber, white meat, red and processed meat, but lower intakes of saturated fat, trans FA, and salt compared with those in the lowest category of HLS.

On micronutrient level, individuals in the highest category of HLS had higher intakes of vitamin A, vitamin C, vitamin E, vitamin B6, potassium, calcium, zinc, selenium, vitamin b12, iron, magnesium, and copper.

On HEI 2010 food groups level, individuals in the highest category of HLS had higher intakes of fruit juice, whole fruit, total vegetables, total protein, legumes, dairy, seafood, and higher FA ratio but lower intake of empty calories. A marginally significant association was revealed between having a higher HLS (HLS = 3) and higher intakes of vitamin D and folate compared to lowest category of HLS (HLS = 1).

### Association between HLS and odds of BC

Multivariable-adjusted odd ratios for BC across categories of HLS is shown in Table 3. Participants with the highest HLS had significantly lower odds of BC (OR: 0.45; 95% CI: 0.31, 0.65) than those with the lowest score. After adjustment for potential confounding variables, participants with the highest HLS were 52% less likely to have BC compared with those with the lowest score (OR: 0.48; 95% CI: 0.32, 0.71). Further adjustment for BMI did not affect our findings; individuals with the highest score were 0.61 times less likely to have BC than those with the lowest score (OR: 0.62; 95% CI: 0.40, 0.94, *P*_*trend*_ = 0.01).

**Table 3:** Multivariable-adjusted ratios for BC across different categories of the Healthy lifestyle (HLS) score

HLS range	1 (*n* = 185)	2 (n = 455)		3 (n = 410)		*P* trend
	OR	OR	95% CI	OR	95% CI	
All population						
Crude	1.00	0.78	0.55–1.11	0.45	0.31–0.65	< 0.001
Model I	1.00	0.85	0.59–1.22	0.48	0.32–0.71	< 0.001
Model II	1.00	0.93	0.63–1.38	0.62	0.4–0.94	0.011
Pre-menopause						
Crude	1.00	1	0.33–2.55	0.63	0.23–1.7	0.278
Model I	1.00	0.98	0.36–2.66	0.67	0.23–1.99	0.386
Model II	1.00	1.05	0.34–3.19	1.59	0.45–5.59	0.478
Post-menopause						
Crude	1.00	0.75	0.51–1.1	0.42	0.28–0.63	< 0.001
Model I	1.00	0.83	0.55–1.23	0.45	0.23–0.69	< 0.001
Model II	1.00	0.91	0.6–1.39	0.56	0.36–0.88	0.004

The association of HLS and BC was also examined stratified by menopausal status (Table 3). Among postmenopausal women, women with the highest HLS had 58% lower odds of BC compared with those with the lowest score (OR: 0.42; 95% CI: 0.28, 0.63, *P*_*trend*_ < 0.001). This inverse association remained significant after further adjustment for potential confounding variables (OR: 0.45; 95% CI: 0.23, 0.69, *P*_*trend*_ < 0.001) and even after controlling for BMI (OR: 0.56; 95% CI: 0.36, 0.88, *P*_*trend*_ = 0.004). We found no significant association between HLS score and odds of BC in premenopausal women in crude or adjusted models.

Model I: Adjusted for age, residence, marital status, SES, education, family history of BC, menopausal status, breast feeding, history of disease and supplement use. Model II: Further controlled for BMI.

### Association between HEI and odds of BC

The association between Healthy Eating Index and breast cancer are shown in Table 4. Participants with the greatest adherence to HEI-2010 recommendations (highest HEI score) had significantly lower odds of BC (OR: 0.40; 95% CI: 0.27, 0.57) than those with the lowest score. After adjustment for potential confounding variables, participants with the healthier diet (highest HEI score) were 60% less likely to have BC compared with those with less healthy diet (lowest HEI score) (OR: 0.40; 95% CI: 0.26, 0.56). This association remained significant even after adjustment for BMI (OR: 0.54; 95% CI: 0.35, 0.82, *P*_*trend*_ < 0.001). When we analyzed data stratified by menopausal status (Table 4), we found that postmenopausal women with the greatest adherence to HEI-2010 (highest HEI score) had 61% lower odds of BC compared with those with the lowest HEI score) (OR: 0.39; 95% CI: 0.26, 0.56, *P*_*trend*_ < 0.001). This result remained significant after further adjustment for potential confounding variables (OR: 0.40; 95% CI: 0.26, 0.62, *P*_*trend*_ < 0.001) and even after controlling for BMI (OR: 0.50; 95% CI: 0.32, 0.79, *P*_*trend*_ = 0.004). Neither in crude nor in adjusted models, was any association between HEI score and odds of BC in premenopausal women.

**Table 4:** Multivariable-adjusted ratios for BC across different categories of the Healthy Eating Index-2010 Score (HEI-2010)

HEI score range	1 (*n* = 254)	2 (*n* = 277)		3 (*n* = 248)		4 (*n* = 271)		P trend
	OR	OR	95% CI	OR	95% CI	OR	95% CI	
All population								
Crude	1.00	0.60	0.42–0.85	0.25	0.17–0.37	0.40	0.27–0.57	< 0.001
Model I	1.00	0.56	0.39–0.81	0.25	0.16–0.38	0.40	0.26–0.56	< 0.001
Model II	1.00	0.60	0.41–0.89	0.33	0.21–0.51	0.54	0.35–0.82	< 0.001
Pre-menopause								
Crude	1.00	0.16	0.05–0.51	0.63	0.26–1.5	0.12	0.12–0.91	0.129
Model I	1.00	0.15	0.04–0.52	0.70	0.26–1.86	0.37	0.11–1.22	0.238
Model II	1.00	0.12	0.03–0.52	1.25	0.38–4.15	1.85	0.40–8.5	0.44
Post-menopause								
Crude	1.00	0.69	0.47–1.01	0.19	0.12–0.31	0.39	0.26–0.56	< 0.001
Model I	1.00	0.66	0.44–0.10	0.19	0.12–0.31	0.40	0.26–0.62	< 0.001
Model II	1.00	0.68	0.45–1.03	0.25	0.15–0.40	0.50	0.32–0.79	< 0.001

Model I: adjusted for age, residence, marital status, SES, education, family history of B. C, menopausal status, breast feeding, history of disease, supplement use, smoking and physical activity. Model II: further controlled for BMI.

### Association between physical activity and odds of BC

Multivariable-adjusted odds ratios for BC across categories of PA are shown in Table 5. The association was also examined by menopausal status. Neither in crude nor in adjusted models, we found no significant association between PA and odds of BC in the whole population as well as in post- or pre-menopausal women.

**Table 5:** Multivariable-adjusted ratios for BC across different categories of Physical activity

PA score range	1 (*n* = 260)	2 (*n* = 264)		3 (*n* = 263)		4 (*n* = 263)		*P* trend
	OR	OR	95% CI	OR	95% CI	OR	95% CI	
All population								
Crude	1.00	1.27	0.88–1.83	1.14	0.79–1.65	1.28	0.89–1.84	0.286
Model I	1.00	1.34	0.91–1.99	1.26	0.85–1.87	1.37	0.93–2.03	0.165
Model II	1.00	1.31	0.87–1.97	1.18	0.78–1.79	1.43	0.95–2.15	0.165
Pre-menopause								
Crude	1.00	2.65	0.97–7.22	1.66	0.57–4.86	2.26	0.78–6.43	0.255
Model I	1.00	3.18	1.08–9.35	1.92	0.61–6.03	2.70	0.87–8.31	0.183
Model II	1.00	5.86	1.61–21.40	3.34	0.86–13.05	4.24	1.15–15.65	0.092
Post-menopause								
Crude	1.00	1.12	0.75–1.66	1.06	0.71–1.58	1.13	0.77–1.68	0.602
Model I	1.00	1.17	0.76–1.80	1.17	0.76–1.80	1.22	0.80–1.86	0.403
Model II	1.00	1.13	0.72–1.77	1.09	0.69–1.70	1.26	0.81–1.96	0.406

Model I: Adjusted for age, residence, marital status, SES, education, family history of B. C, menopausal status, breast feeding, history of disease, supplement use, smoking and HEI score. Model II: Further controlled for BMI.

### Association between smoking and odds of BC

Multivariable-adjusted odds ratios for BC across categories of smoking are shown in Table 6**.** Smokers were 41% more likely to have BC than non-smokers (OR: 1.41; 95% CI: 0.99, 2.01, *P*_*trend*_ = 0.055). However, after adjustment for potential confounding variables, this association disappeared. Neither in crude nor in adjusted models, we found no significant association between smoking and BC in pre- or postmenopausal women.

**Table 6:** Multivariable-adjusted ratios for BC across different categories of Smoking

Smoking Categories	Non-smoker	Smoker		P trend
	OR	OR	95% CI	
All population				
Crude	1.00	1.41	0.99–2.01	0.055
Model I	1.00	1.23	0.84–1.82	0.286
Model II	1.00	1.01	0.67–1.52	0.943
Pre-menopause				
Crude	1.00	0.57	0.12–2.64	0.474
Model I	1.00	0.47	0.09–2.53	0.411
Model II	1.00	0.47	0.08–2.71	0.384
Post-menopause				
Crude	1.00	1.40	0.97–2.03	0.073
Model I	1.00	1.31	0.87–1.98	0.193
Model II	1.00	1.08	0.70–1.65	0.726

Model I: Adjusted for age, residence, marital status, SES, education, family history of B. C, menopausal status, breast feeding, history of disease, supplement use, HEI score and physical activity. Model II: Further controlled for BMI.

## Discussion

In this case-control study, we found a significant inverse association between adherence to HLS as well as HEI-2010 and odds of BC. This association remained significant after adjusting for several confounding variables. In our stratified analysis by menopausal status, the association remained significant among postmenopausal women, while no association was found among premenopausal group. In terms of components of HLS, no association was found between smoking and PA and odds of BC. To our knowledge, this is the first study to investigate the association between HLS and the risk of BC in a Middle Eastern country.

BC is the most prevalent cancer among women worldwide. Healthy lifestyle modification is an important factor in BC prevention. Earlier studies have shown that at least 25 to 30% of BC cases could be prevented if healthy lifestyle was chosen [21]. In the current study, we found that adhering to HLS was associated with a reduced odd of BC. Our findings were in line with previous reports that found an inverse association between HLS and BC. In the Women’s Health Initiative Study, postmenopausal women in the highest quintile of Healthy Lifestyle Index (HLI) had a 30% decreased risk of BC compared to women in the lowest quintile (HR: 0.70; 95% CI: 0.64–0.76). In addition, a 4% decreased risk of BC was found per unit increase in HLI score (HR: 0.96; 95% CI: 0.95–0.97) [21]. In the EPIC study, there was a 3% reduction in the risk of BC per point increase of the Healthy Lifestyle Index Score (HLIS) among post-menopausal women [22]. In addition, Gemert et al. showed that modifiable risk factors were directly responsible for about one out of four postmenopausal BC cases. Modifiable risk factors composed of BMI, physical inactivity, alcohol consumption, smoking and low dietary fiber intake [23]. In the E3N cohort, adherence of postmenopausal women to a healthy lifestyle by following the recommendations for smoking, BMI, alcohol consumption, fruit and vegetable consumption, and PA, prevented BC by 6.3% [24]. However, an increased risk of breast cancer-specific mortality was found for individuals with healthy behavior index compared to those with a lower healthy lifestyle score, however, the results was not statistically significant [14]. Therefore, it seems that adherence to healthy lifestyle could be used as a good preventive measure to reduce BC.

When we analyzed the data by menopausal status, we found a significant inverse association in postmenopausal women, but not in premenopausal women. The same associations were found in some studies [21–24]. However, some studies have found a significant inverse association among premenopausal women as well [13, 25]. In a case-control study from Morocco, Khalis et al. found a significant association in premenopausal women. However, the same associations were also reached in postmenopausal women [25]. Furthermore, a study from Mexico revealed that HLI was associated with a decreased odds of BC among both pre- and postmenopausal women [13].

One might question why we only included diet, PA, and smoking as lifestyle factors and did not include other factors in the HLS construction. Earlier studies on lifestyle score have mostly used the variables we used in this study. However, some studies have also included BMI in the score, but as being overweight or obese is a result of an unhealthy lifestyle, we preferred not to include it in the score. We did not consider hormone use because it is not part of lifestyle, rather it’s a medication use to control hormone levels. In addition, alcohol consumption can be considered in the diet part of the scoring but given the culture of the study population and their religion, alcohol consumption is forbidden in the country and we did not collect information on this variable. With regards to breastfeeding and its history among study population, we believe that despite its contribution to the risk of BC, it is not a lifestyle factor. In general, lifestyle constitutes a combination of diet, physical activity, smoking and stress. However, we did not have information on stress in the current study population.

Lack of finding a significant association between HLS and odds of BC in premenopausal women might be explained by the low number of premenopausal women in the current study (850 post- vs 200 premenopausal women). Another explanation is the variation in components of HLS. In the study by Khalis et al., they used an index composed of diet, PA, BMI, smoking, alcohol consumption, and breastfeeding [25]. In the study by Sánchez-Zamorano et al., their index identified healthy lifestyle as being in the lowest tertile of the Western dietary pattern, never consuming alcohol, smoking less than 100 cigarettes, and practicing moderate and vigorous intensity exercise [13]. We used HEI as a component of healthy diet, while most other studies have used fruit and vegetables as the healthy diet.

There are several mechanisms that explain the inverse association between healthy lifestyle and odds of BC. Healthy diet plays an important role in cancer prevention as shown in the literature. Hormone receptor-positive breast tumor depends on hormonal risk factors for ongoing proliferation; while hormone receptor-negative BC depends more on non-hormonal risk factors like diet [26]. A healthy diet that is abundant in dietary fiber, antioxidants and vitamins might decrease the proliferation of ER negative BC through suppressing the inflammatory response, neutralizing free radicals and preventing DNA damage. Another plausible mechanism is through weight management, since adiposity have been defined as a risk factor for BC [27]. PA and nonsmoking may also contribute to the protective associations of HLS with chronic conditions; however, their role in the reduced odds of BC in our study was not prominent.

A healthy lifestyle composing of a healthy diet along with regular PA and nonsmoking behavior has been shown to decrease the risk of BC. Despite lack of finding significant associations between diet and BC in most previous studies, we found a significant inverse association between adhering to a healthy diet and odds of BC. The main difference between our study and previous studies is using HEI-2010 as an index of healthy diet, while other studies have mostly used some components of the healthy diet instead. Another possible explanation for the difference between our findings and those in previous studies might be attributed to the lack of alcohol consumption in Iranian’s society. One additional point that should be considered is high consumption of low fat, rather than high-fat dairy products in Iran compared with other countries. We did not find any significant association between smoking and BC in the current study, in contrast to the results of previous studies. In fact, prevalence of smoking in Iranian women is not so high compared to other countries. Based on Tehran Lipid and Glucose Study (TLGS), the prevalence of smoking in Iranian women is 2.1% [28]. In addition, we did not find any significant association between PA and BC, though previous studies have shown a significant inverse association between PA and BC. One possible explanation is that most Iranian women are housewives, which might lead to have low PA levels. Meanwhile, the Iranian national study have shown that the prevalence of inactivity in urban and rural areas of women aged 15 years and older was 58.8% [29]. Given the high prevalence of inactivity, the distribution of this variable might not be normal to let us find a real association. In addition, the IPAQ used in the assessment of PA in the current study might have some limitations, though it was validated in Iran [30].

Our study has several strengths. We used the HEI-2010 to assess dietary quality, rather than using some components of the diet. In addition, we accounted for several confounding factors in our analysis. The use of validated FFQ and being the first study examining the link between HLS and odds of BC in a Middle Eastern population would be among others. However, our study has some limitations. The case-control design of the study, which is subject to selection and recall bias, prohibited us to confer causality. Measurement errors might result in misclassification of study participants in terms of dietary intakes. The number of postmenopausal women is greater than the number of premenopausal women. Moreover, HEI-2010 is a USA-based index for assessing diet quality which might not be totally applicable to a population with a different diet and lifestyle. Hormone receptor status is very important in studying factors related to BC as evidenced by previous studies, yet we did not have information about it in the current study which might be an important limitation.

While previous studies in Iran have assessed the association between individual lifestyle factors and BC risk, no study was done before examining the association between a combined healthy lifestyle score and odds of BC. In addition, limited studies have used HEI-2010 as a dietary assessment tool in their lifestyle score. Our study contributed to the accumulating evidence for adherence to a high HLS or HEI-2010 as being associated with decreased BC odds in women in general.

## Conclusion

In conclusion, we found a protective association between HLS and odds of breast cancer. This association was particularly seen in postmenopausal women. Therefore, adoption to a healthy lifestyle, including healthy diet, PA and nonsmoking behavior might help prevent the prevalence of breast cancer in the community setting. No significant association was found between PA or non-smoking behavior with odds of BC. Further studies, however, are needed to confirm our current findings.

## Data Availability

The datasets used and/or analyzed during the current study are available from the corresponding author on reasonable request.

## Abbreviations

ANOVA: One-way analysis of variance
BC: Breast Cancer
BMI: body mass index
CI: Confidence Interval
FFQ: food frequency questionnaire
HEI-2010: Healthy Eating Index-2010
HLS: Healthy Lifestyle Score
MUFAs: monounsaturated fatty acids
OR: Odd Ratio
PA: Physical Activity
PUFAs: polyunsaturated fatty acids
SD: Standard Deviation
SES: Social economic status
